# In-depth Analysis of IS*6110* Genomic Variability in the *Mycobacterium tuberculosis* Complex

**DOI:** 10.3389/fmicb.2022.767912

**Published:** 2022-02-24

**Authors:** Jessica Comín, Isabel Otal, Sofía Samper

**Affiliations:** ^1^Unidad de Investigación Traslacional, Hospital Universitario Miguel Servet, Instituto Aragonés de Ciencias de la Salud, Zaragoza, Spain; ^2^Fundación IIS Aragón, Zaragoza, Spain; ^3^Facultad de Medicina, Universidad de Zaragoza, Zaragoza, Spain; ^4^CIBER de Enfermedades Respiratorias, Madrid, Spain

**Keywords:** tuberculosis, IS*6110*, *Mycobacterium tuberculosis* complex, IS*6110* genomic variability, tuberculosis evolution

## Abstract

The insertion sequence (IS) *6110* is a repetitive mobile element specific for the *Mycobacterium tuberculosis* complex (MTBC) used for years to diagnose and genotype this pathogen. It contains the overlapping reading frames *orfA* and *orfB* that encode a transposase. Its genetic variability is difficult to study because multiple copies are present in the genome. IS*6110* is randomly located, nevertheless some preferential locations have been reported, which could be related to the behaviour of the strains. The aim of this work was to determine the intra- and inter-strain genetic conservation of this element in the MTBC. For this purpose, we analysed 158 sequences of IS*6110* copies from 55 strains. Eighty-four copies were from 17 strains for which we knew all the locations in their genome. In addition, we studied 74 IS*6110* copies in 38 different MTBC strains in which the location was characteristic of different families including Haarlem, LAM, S, and L6 strains. We observed mutation in 13.3% of the copies studied and we found 10 IS*6110* variants in 21 copies belonging to 16 strains. The high copy number strains showed 6.2% of their IS*6110* copies mutated, in contrast with the 31.1% in the low-copy-number strains. The apparently more ancient copy localised in the DR region was that with more variant copies, probably because this was the most studied location. Notably, all Haarlem and X family strains studied have an IS*6110* in *Rv0403c*, suggesting a common origin for both families. Nevertheless, we detected a variant specific for the X family that would have occurred in this location after the phylogenetic separation. This variant does not prevent transposition although it may occur at a lower frequency, as X strains remain with low copy number (LCN) of IS*6110*.

## Introduction

The insertion sequence (IS) *6110* has been described as a repetitive mobile element specific for the *Mycobacterium tuberculosis* complex (MTBC; Thierry et al., 1990). It has been used for years to diagnose and to characterise at the molecular level *M. tuberculosis* strains by the IS*6110*-restriction fragment length polymorphism (RFLP) technique (Thierry et al., 1990; Brisson-Noel et al., 1991; Otal et al., 1991). It is 1,355 bp and is flanked by inverted repeats (IR) of 28 bp (Thierry et al., 1990). When transposition occurs, it creates a duplication of 2–4 bp in the insertion site (Mendiola et al., 1992; Dale, 1995). IS*6110* contains two overlapping reading frames called *orfA* and *orfB*. While the product orfAB is a transposase, the individual products orfA and orfB inhibit transposition, so the proportion of each product is important for the autoregulation of transposition (Sekine et al., 1997). Transcription of this element is regulated by a-1 frameshift due to a ribosomal slippery sequence and by a pseudoknot in the mRNA; these processes lead to a transposition based on a copy-out-paste-in mechanism (Gonzalo-Asensio et al., 2018).

The global *M. tuberculosis* population can be divided into several phylogeographic lineages (Ls) and families. The genetic diversity found in circulating strains plays an important role in disease outcome. The number of IS*6110* copies in the genome is variable and seems to be related to the evolution of the strains. Modern Ls have more IS*6110* copies than ancient Ls, and they are considered to be better adapted to high-density populations (Gonzalo-Asensio et al., 2018). It seems that if the IS*6110* is inserted in a transcriptionally active region of the genome, the number of copies increases, so the variable number of copies present in different strains could be due to the genomic region in which they are inserted, although a large number of IS*6110* copies may be deleterious for the strain because they can remove parts of the genome (Wall et al., 1999).

While IS*6110* supposedly transposes randomly, there are some preferential location sites such as the CRISPR region, also known as the DR region in *M. tuberculosis*; it was probably the first IS copy because it is present in almost all MTBC isolates (Hermans et al., 1991). Other hot spots described are: the *plcD* gene (Vera-Cabrera et al., 2001), members of the PPE family genes (Sampson et al., 1999; Beggs et al., 2000), the intergenic region *dnaA*:*dnaN* (Supply et al., 2006), and other ISs (Fang and Forbes, 1997; Fang et al., 1999). In addition, characteristic locations of IS*6110* in different Ls have been observed (Roychowdhury et al., 2015). The function of IS*6110* remains unknown although some general effects have been described. The first is the disruption of the gene in which it has been inserted, with a possible deleterious effect (Sampson et al., 1999; Beggs et al., 2000; Warren et al., 2000; Yesilkaya et al., 2005). The second is the recombination and the deletion or inversion of the DNA in between, a phenomenon that can occur when there are two IS relatively close to each other (Sampson et al., 2003). A third effect is that IS*6110* can act as a promoter of the downstream gene (Beggs et al., 2000; Safi et al., 2004; Soto et al., 2004).

For the MTBC, the rate of point mutations has been determined to be 1 × 10^−9^ events per site per generation. However, it has been demonstrated that the rate of point mutations for the IS*6110* is about 7.9 × 10^−5^, higher compared with the rest of the genome, indicating that IS*6110* is under selective selection (Tanaka, 2004). There are few studies focused on the sequence genetic variability of this element. Dale et al. (1997) amplified and sequenced IS*6110* of four *M. tuberculosis* strains, each carrying one copy, and one of the copies carried by the Beijing W strain, which has 17 copies. They compared the sequences with the Bacillus Calmette and Guérin (BCG) copy published and found they are identical. Nevertheless, they only checked one copy of each strain. Conversely, Thabet et al. (2015) found several different haplotypes when they randomly cloned and sequenced only one of the IS*6110* copies of some characteristic strains from their region.

In this work, we aimed to determine whether IS*6110* is a conserved gene, as Dale et al. (1997) suggested or, on the contrary, it is relatively common to find this IS mutated, as Thabet et al. (2015) suggested. In addition to the clinical relevance, since it is widely used for TB diagnosis, we thought mutations of the IS*6110* could affect its transposition ability. Therefore, we planned to find out the genetic variability of the sequence. For this purpose, we applied two different approaches. The first was the study of all the IS*6110* copies present in the same strain to determine whether they had an identical sequence. For the second approach, a sample collection was used to study IS*6110* copies in different MTBC families for which the location had been previously published (Reyes et al., 2012; Comín et al., 2021) to elucidate how conserved IS*6110* is among families for the same location.

## Materials and Methods

### Collection of Samples

#### Study of IS*6110* in Available Completed Genomes

We selected the available MTBC complete genomes from the NBCI database. First, we checked the entire sequence of the 16 IS*6110* copies of H37Rv (NC_000962.3) using the Tuberculist BLAST tool.^1^ Second, we looked for the IS*6110* copies of CDC1551 (AE000516.2), *Mycobacterium africanum* (CP010334.1 and FR878060.1), *Mycobacterium bovis* (LT708304.1), and *M. bovis* BCG (CP033311.1, CP014566.1, and AM408590.1) loaded in the NCBI database. We checked the insertion points using Tuberculist. All the points detailed in the results refer to the H37Rv genome, unless otherwise indicated (Table 1).

**Table 1:** Summary of IS*6110* copies analysed in the strains for which the location of all the IS copies within their genomes is known, detailing the IS copies mutated by comparison with the reference IS in *Mycobacterium tuberculosis* H37Rv.

Strain	Lineage (Family)	CN strain	IS analysed (*N* = 84)	Mutated copies location	Mutation detected	Non-mutated copies location	Reference
H37Rv	L4.9	HCN	16	-	-	all wt	NC_000962
CDC1551	L4.1.1.3	LCN	4	*Rv0403c,cut,ppe46*	*orfB Gly215Ser* ^*^	*DR region*	AE000516.2
*M. africanum* 25	L6	LCN	5	*Rv3750c*	*orfB gap in 110n*.	all wt but one	*CP010334.1*
*M. africanum* GM041182	L6	HCN	7	DR region	orfA syn 33 aa	all wt but one	FR878060.1
*M. bovis* AF2122/97	L Animal	LCN	1	-	-	all wt	LT708304.1
*M. bovis BCG_S49*	L Animal	LCN	1	-	-	all wt	CP033311.1
*M. bovis BCC_Tokyo*	L Animal	LCN	2	-	-	all wt	CP014566.1
*M. bovis BCG_Pasteur*	L Animal	LCN	1	-	-	all wt	AM408590.1
*M. tuberculosis* MtZ	L4.8	HCN	12	-	-	all wt	Isabel Millan-Lou et al., 2013
*M. tuberculosis* GC1237	L2	HCN	18	-	-	all wt	Alonso et al., 2011
*M. bovis* B	L Animal	LCN	2	-	-	DR region, Rv0756c:phoP	Sagasti et al., 2016
HMS 2382	L6	LCN	3	DR region	orfB Asp2Gly	moaX,Rv0963c	Comín et al., 2021
HMS 2407	L6	LCN	3	-	-	lipX:mshB,moaX, DR region	Comín et al., 2021
HCU 3445	L4	LCN	1	-	-	DR region	
HMS 2485	L4.1.1.3	LCN	2	*Rv0403c*	*orfB Gly215Ser* ^*^	DR region	
HCU 3717*	L4.1.1.3	LCN	4	DR region (Otal et al., 1991), *Rv0403c*, and *MT1802:cut1*	*orfB Gly215Ser* ^*^		Comín et al., 2021
HMS 2445	L4.1.1.3	LCN	2	*Rv0403c*	*orfB Gly215Ser* ^*^	DR region	

Even though HCU3717 strain only has four out of five IS copies analysed, it has been included in this section because the fifth copy is located in ppe46 gene (not successfully amplified). CN, Copy number; HCN, high copy number; and LCN, low copy number. *Mutation associated to X family.

#### Strains Used for Sequencing IS*6110*

All the DNAs used in this work were extracted from clinical *M. tuberculosis* isolates previously genotyped by IS*6110*-RFLP and spoligotyping in our laboratory, and stored at −20°C. Strains previously characterised and with all their IS*6110* copies located were selected to amplify all the copies within the genome. These strains were: *M. tuberculosis Zaragoza* (*MtZ*, L4; Isabel Millan-Lou et al., 2013), GC1237 (L2; Alonso et al., 2011), and seven low copy number (LCN) of IS*6110* strains belonging to different lineages. These LCN strains were HMS 2407 and HMS 2382 (L6), HCU 3445 (L4), HMS 2484, HMS 2445, HCU 3717 (Comín et al., 2021; L4 strains belonging to X family), and *M. bovis* B (hypervirulent strain; Sagasti et al., 2016; Table 1). For the extension of the study (Table 2), we used 32 L4 strains distributed in different families: 10 LAM strains (HMS 18005, HSJ 241, HMS 18045, HMS 18017, HMS 18025, HMS 18010, HMS 18047, HMS 18048, HMS 18018, and ara217, a LAM9 strain studied for producing an outbreak in our region; Comin et al., 2020), 12 Haarlem strains (HSJ 238, HMS 18009, HMS 18021, HMS 18037, HCU 3729, HMS 18031, HMS 18022, HMS 18007, HMS 18046, HSJ 234, HMS 18001, and HMS 18002), one S strain (HMS 18019), and seven T strains (HCU 3718, HMS 18041, HMS 18035, HMS 18042, HMS 18014, HMS 18040, and HMS 18044). In addition, we also included one L3 strain belonging to the CAS1_DELHI family (HMS 18038) and seven *M. africanum* L6 strains (HCU 2828, HCU 3775, HMS 14017, HMS 2000, HMS 1942, HSJ 66, and HMS 1693; Table 2).

**Table 2:** Summary of the IS*6110* sequences localised in preferential sites published for determined *Mycobacterium tuberculosis* families (Reyes et al., 2012; Comín et al., 2021).

Strain	Lineage	Family by SIT	CN STRAIN	Number of IS*6110* ^*^/studied (*N* = 74)	Mutated copies location	Mutation detected	Non-mutated copies location
HSJ 238	L4.1.2.1	Haarlem3	HCN	11/3	*-*	*-*	*Rv1754c,Rv0403c,Rv0963c*
HMS 18009	L4.1.2.1	Haarlem3	HCN	10/2	*DR region*	*orfB Asp2Gly*	*Rv0403c*
HMS18021	L4.1.2.1	Haarlem3	HCN	12/2	*-*	*-*	*Rv0403c,Rv0963c*
HMS 18037	L4.1.2.1	Haarlem3	HCN	11/4	*Rv0963c*	*IR*	*Rv1754c,Rv2336*, and *Rv0403c*
HCU 3729	L4.1.2.1	Haarlem3	HCN	7/5	*-*	*-*	*Rv1754c,Rv2336,Rv0403c,Rv0963c*, and *DR region*
HMS 18031	L4.1.2.1	Haarlem3	HCN	8/2	*Rv0963c*	*orfB syn aa174*	*Rv0403c*
HMS 18022	L4.1.2.1	Haarlem1	LCN	6/4	-	-	*Rv1754c,Rv2336,Rv0403c*, and *Rv0963c*
HMS 18007	L4.1.2.1	Haarlem1	HCN	10/1	-	-	*DR region*
HMS 18046	L4.1.2.1	Haarlem1	HCN	9/4	*DR region*	*orfB Asp2Gly*	*Rv0963c,Rv1754c*, and *Rv0403c*
HSJ 234	L4.1.2.1	Haarlem3	HCN	11/3	*Rv0963c*	*orfB syn103*	*Rv2336,Rv0403c*
HMS 18001	L4.1.2.1	Haarlem1	HCN	8/4	*-*	*-*	*Rv1754c,Rv0403c,Rv0963c*, and *DR region*
HMS 18002	L4.1.2.1	Haarlem1	HCN	10/4	*-*	*-*	*Rv1754c,Rv2336,Rv0963c*, and *DR region*
HMS 18005	L4.3	LAM3	LCN	5/1	*-*	*-*	*Rv3113*
HSJ 241	L4.3	LAM3	HCN	15/2	*-*	*-*	*Rv3113,DR region*
HMS 18045	L4.3	LAM3	HCN	15/2	*-*	*-*	*Rv3113,DR region*
HMS 18017	L4.3	LAM12	HCN	14/2	*-*	*-*	*lpqQ:Rv0836c,DR region*
HMS 18025	L4.3	LAM9	HCN	11/2	*lpqQ:Rv0836c*	*orfB Gly123Arg*	*DR region*
HMS 18010	L4.3	LAM9	HCN	14/2	-	-	*Rv1754c,DR region*
HMS18047	L4.3	LAM4	HCN	14/1	-	-	*DR region*
HMS 18048	L4.3	LAM9	HCN	10/3	-	-	*Rv1754c,lpqQ:Rv0836c*, and *DR region*
HMS 18018	L4.3	LAM3	HCN	12/3	-	-	*Rv1754c,MT3426:MT3427*, and *lpqQ:Rv0836c*
Ara217**	L4.3	LAM9	HCN	13/2	-	-	*MT3426:MT3430,Rv1754c:cut1*
HMS 18019	L4.4.1.1	S	HCN	11/1	-	-	*pks9*
HCU 3718	L4	T1	HCN	9/1	-	-	*DR region*
HMS 18035	L4	T2	LCN	5/1	-	-	*DR region*
HMS 18040	L4	T5	HCN	8/1	-	-	*DR region*
HMS 18042	L4	T2	HCN	7/1	-	-	*DR region*
HMS 18041	L4	T1	HCN	9/1	-	-	*DR region*
HMS 18044	L4	T5_MAD2	LCN	6/1	-	-	*DR region*
HMS 18014	L4	T5_MAD2	LCN	6/1	*DR region*	*orfA Thr67Iso*	-
HMS 18038	L3	CAS1_DELHI	HCN	16/1	-	-	*DR region*
HCU 2828	L6	*AFRI_1*	HCN	8/1	-	-	*lipX:mshB*
HCU 3775	L6	*AFRI_1*	LCN	3/1	-	-	*lipX:mshB*
HMS 1693	L6	*AFRI_1*	LCN	3/1	*lipX:mshB*	*IR*	-
HMS 14017	L6	*AFRI_1*	LCN	3/1	*lipX:mshB*	*IR*	-
HMS 1942	L6	*AFRI_1*	LCN	unk/1	-	-	*moaX*
HMS 2000	L6	*AFRI_1*	LCN	5/1	-	-	*moaX*
HSJ 66	L6	*AFRI_1*	LCN	3/1	-	-	*moaX*

*CN, Copy number; HCN, high copy number; LCN, low copy number; and SIT, Spoligo International Type*.

* *Total number of IS6110 based in the IS6110 RFLP pattern*.

** *Outbreak strain (Comin et al., 2020)*.

### Analysis of the IS*6110* DNA Sequences

#### Amplification and Sequencing of the Region Including the IS*6110*

For the PCR, MyTaq DNA polymerase (Lot No: MT-7171128, Bioline) and 5x MyTaq Reaction Buffer (Lot No: MTB-T17212A, Bioline) were used. We performed an initial denaturation at 95°C for 1 min. Then we performed 35 cycles: denaturation at 95°C for 15 s, annealing at the primer temperature for 15 s, and extension at 72°C for 30 s. The specific primers used for each point of insertion are shown in Supplementary Tables S1–S4. We used a commercial kit (GFX PCR DNA and Gel Band Purification Kit, GE Healthcare, Lot: 16834032) to clean the samples before sequencing. We used the same kit when we needed to extract the DNA from a gel band. The samples were sequenced using capillary electrophoresis with the specific primers flanking the different IS copies and the internal IS primers: IS7 (TTCGGACCACCAGCACCTAACC), IS9 (GCTTTGCCGCGGGTGG), and IScom (ATGTCAGGTGGTTCATCGAGGAGG) in a 3500XL Genetic Analyser (Applied Biosystem).

#### Study of the Sequences

The sequences obtained were studied using the BioEdit program (Informer Technologies, Inc.), a biological sequence alignment editor. We also used Tuberculist^1^ and Bovilist,^2^ where the annotation of *M. tuberculosis* H37Rv and *M. bovis* AF2122/97 can be found. We also used the BLAST tool from the NCBI^3^ to study CDC1551, two *M. africanum* strains, and three *M. bovis* BCG strains. Integrative Genomics Viewer (IGV; Robinson et al., 2011) software, which allows the study of the whole genome in an interactive way, was also used.

## Results

**Figure 1:**
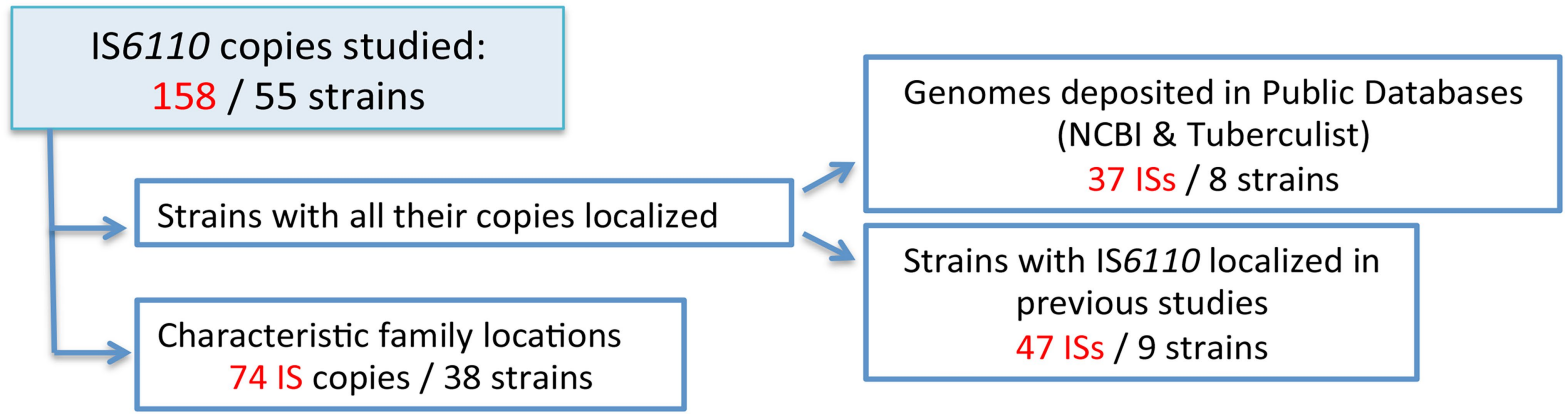
Summary of the different IS*6110* copies studied in this work.

We aimed to determine the genetic variability of the IS*6110* sequence. With this purpose, a large number of copies were analysed. The first approach of the study focused on the comparison of all the IS copies present in a strain to determine whether different copies coexist in the same genome. Initially, we analysed the strains whose genomes were available in the databases. Furthermore, we studied several strains whose IS*6110* locations were known as result of our previous studies. For the second approach, we studied IS*6110* in specific locations defined for different families, including Haarlem, LAM, S, and L6 strains (Reyes et al., 2012; Comín et al., 2021) to determine its inter- and intra-strain variability using a sample collection. In addition, CAS_DELHI and T strains were included to study the IS copy located in the DR region. As a result, 158 IS*6110* sequences from 55 different strains were analysed (Figure 1). Twenty-one of the copies, belonging to 16 strains, had a mutation (13.3%). Among the high copy number strains, seven out of the 113 copies studied (6.2%) were mutated while 14 out of the 45 copies (31.1%) showed a mutation in the LCN strains.

### Review of the Different IS Copies in the NCBI and Tuberculist Databases

To investigate the IS*6110* sequence variability, we checked MTBC strains whose genomes are available in the NCBI and Tuberculist databases: *M. tuberculosis* H37Rv and CDC1551, *M. africanum* 25 and GM041182, *M. bovis* AF2122/97, and three different BCG strains. The results are summarised in Table 1.

Initially, the analysis of the 16 IS*6110* copies registered in Tuberculist belonging to the H37Rv strain showed that all the sequences are identical to each other. Therefore, we chose the first copy in its genome, from points 889,020 to 890,375 (*Rv0795* and *Rv0796*), as the reference wild-type IS for the successive comparisons.

Searching the IS*6110* copies in the different genomes available in the NCBI database showed several IS copy variants. The CDC1551 strain has four IS*6110* copies, located in *Rv403c* [point 483,296, reverse direction (−)], *cut1* (1,989,058, −), DR region (3,120,523:3121879, −), and *ppe46* genes [3,377,327, forward direction (+)]. Only the copy in the DR region is wild type, while the other three IS*6110* copies share a mutation in the first base of codon 215, within *orfB* of the IS sequence (G/A, Gly/Ser).

We performed the same procedure for *M. africanum* 25. We found five copies of IS*6110* in its genome, inserted in *lipX:mshB* (1,300,194, −), *Rv1765c* (1,998,416, +), DR region (3,120,523:3,121,879, −), *moaX* (3,709,622, −), and *Rv3750c* genes (4,198,431, +). Only the copy in *Rv3750c* showed a gap in nucleotide 110 of *orfB*. *Mycobacterium africanum* GM041182 has seven IS*6110* copies. Five of them are at the same location and direction as those in strain 25. Nevertheless, the one located in the DR region has a synonymous mutation in the last base of codon 33 (A/G), within *orfA* of the IS sequence. The previous gap found in strain 25 was not detected. The sixth and seventh copies are inserted in *pks8* (1,882,012, +) and *Rv3128c*:*Rv3129* genes (3,494,393, −), both presenting the wild-type sequence.

The single IS*6110* copy of *M. bovis* AF2122/97, inserted in the DR region (3,120,523–3,121,879, −), is wild type. We chose three BCG strains, CP033311.1, CP014566.1, and AM408590.1. All of them have the copy of the DR region inserted at the same point and direction as *M. bovis* AF2122/97. A second copy of CP014566.1 is upstream the *phoP* gene (851,590, −). All the copies are wild type.

### Analysis of the IS*6110* in Clinical Strains With All Their Copies in Known Locations

**Figure 2:**
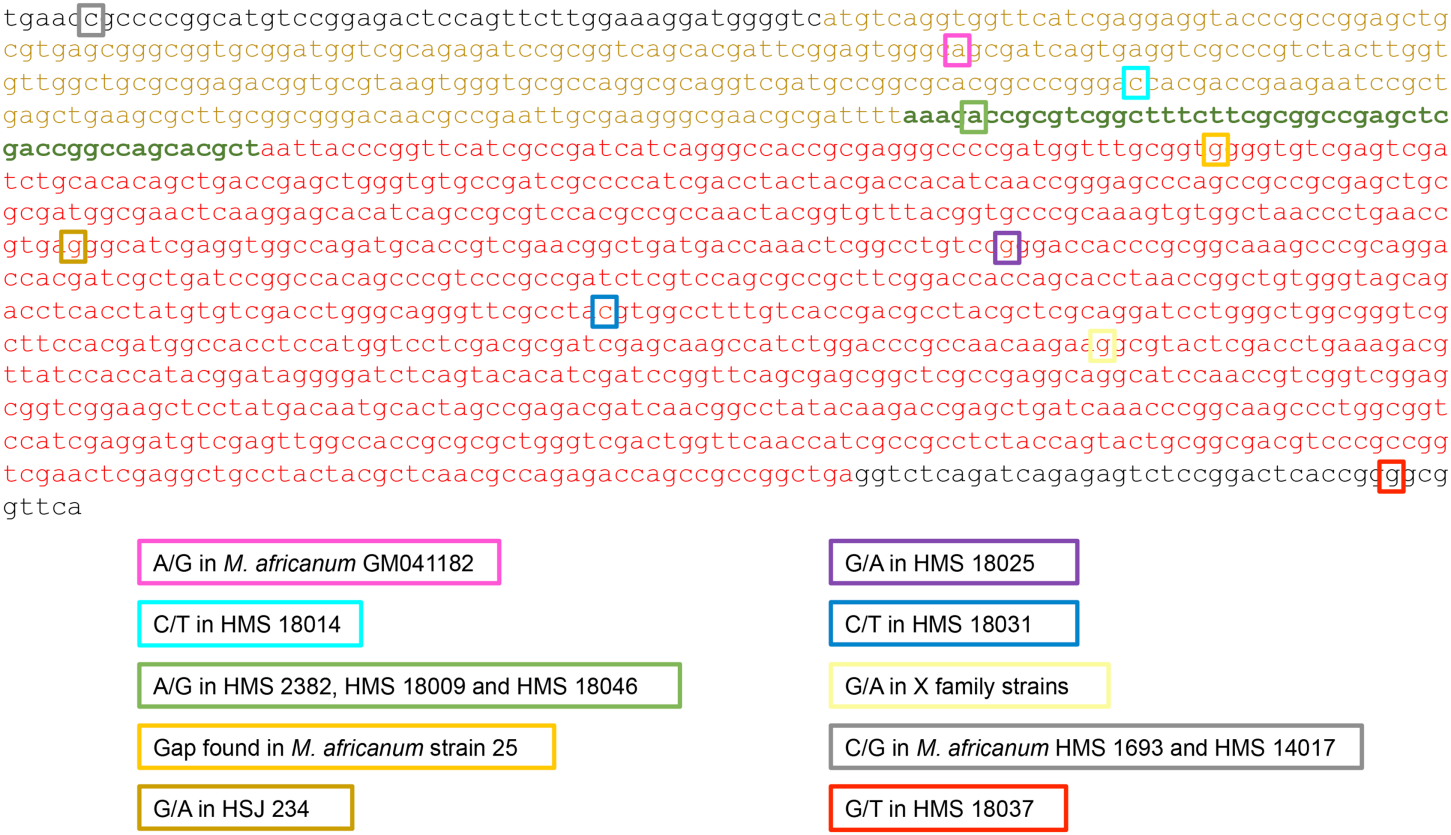
Sequence of IS*6110*, highlighting in different coloured squares the points, the nucleotide changes, and the different strains where the 10 mutations were found. *orfA* is written in yellow, *orfB* is in red, and the overlapping region is in green. The black letters at the ends represent the inverted repeats.

Using the information obtained in previous studies by our group, we were able to successfully amplify and sequence 47 IS*6110* copies from the genomes of three outbreak strains previously characterised (MtZ, GC1237, and *M. bovis* B) and six LCN strains (HMS 2382, HMS 2407, HCU 3445, HMS 2485, HMS 2445, and HMS 3717). The specific characteristics of these strains can be found in Table 1. The 12 IS*6110* copies of the *MtZ* strain (locations can be found in Supplementary Table S1), the 18 copies of the GC1237 strain (Supplementary Table S2), and the two copies of *M. bovis* B present the wild-type sequence. Among the analysed sequences of the LCN strains, we found two different mutations. *Mycobacterium africanum* HMS 2382 has a change in the overlapping region of *orfA* and *orfB* in its IS*6110* copy within the DR region (−). This mutation (A/G) affects the second amino acid (Asp/Gly) after the ribosomal slippery. We also found a mutation in the IS*6110* copies inserted at *Rv0403c* (−) in HMS 2485, HMS 2445, and HCU 3717 strains, all belonging to X family. This Gly/Ser (G/A) mutation coincides with that found in CDC1551 IS copies (which also belongs to X family). This mutation was also found in three other IS copies of HCU 3717, located in *MT1802:cut1* and the DR region (two copies). We were not successful in the amplification of its fifth copy, located in *ppe46*, but if it was also mutated, HCU 3717 would be the unique strain with all its IS copies mutated. All the other copies from the LCN strains analysed are wild type. The exact point of the mutations found is detailed in the sequence of IS*6110* in Figure 2.

### Analysis of the IS*6110* Sequences in Characteristic Locations in MTBC Families

To extend the study to other MTBC families and to more strains, we relied on the specific IS*6110* locations for Haarlem, LAM, S, and L6 strains detailed in other publications (Reyes et al., 2012; Comín et al., 2021). For the hot spot DR location, we included additional T and CAS_DELHI isolates. The results are shown in Table 2.

According to Reyes et al. (2012), 96% of Haarlem strains have an IS inserted in *Rv0403c* (483,296, −), *Rv2336* (2,610,861, +), and *Rv1754c* genes (1,986,622, +), and 89% have a copy in *Rv0963c* (1,075,948, −). We succeeded in the amplification of 10 IS*6110* copies in *Rv0403c* in different strains, five copies in *Rv2336* and seven copies in *Rv1754c*. All of them are wild type. However, we found two different synonymous mutations for the IS copy in *Rv0963c* in two of the 10 strains analysed. HMS 18031 has a mutation in the last base of codon 174 and HSJ 234 has a mutation in the last base of codon 103, both in *orfB*. The rest of the copies are identical to the reference IS sequence.

Regarding the LAM family, it has been reported (Reyes et al., 2012) that more than 96% strains have an IS inserted in *Rv1754c* (1,986,623, −), more than 95% have it inserted in *lpqQ:Rv0836c* (932,202, −), and more than 90% have it inserted in *Rv3113* (3,480,371, +). Based on these findings, we analysed four sequences from the IS inserted in *Rv1754c*, four sequences from the IS inserted in *lpqQ:Rv0836c*, and three sequences from the IS inserted in *Rv3113*. All the sequences are wild type, except the one in *lpqQ:Rv0836c* of HMS 18025, which has a mutation in the first base of codon 123 after the ribosomal slippery sequence (*orfB*, Gly/Arg). Furthermore, we studied one more location in *MT3426:MT3430* in the context of a LAM strain outbreak investigation (ara217; Comin et al., 2020), successfully amplified for this strain. We also studied this location for the other LAM strains but it was only successfully amplified for one of them. Both present the wild-type genotype.

According to Reyes et al. (2012), more than 85% of S strains have an IS*6110* copy in the *pks9* gene (1,889,066, −). We studied the IS*6110* copy inserted in this location in one S family strain (HMS 18019). This copy is identical to the reference IS sequence.

A recent work (Comín et al., 2021) determined that 100% of the L6 strains have a copy of IS*6110* in the *moaX* gene (3,709,622, −). We analysed five copies for this position, all presenting the wild-type genotype. In the same work, the authors reported that it is common to find a copy between the *lipX* and *mshB* genes (1,300,194, −) for L6. We successfully amplified four copies for this location, each showing the wild-type *orfA* and *orfB* genes.

Almost all MTBC strains have an IS*6110* inserted in the DR region. We amplified the IS of this region for 30 strains. We found three different mutations in five strains (Figure 2). HMS 2382 (L6), HMS 18009, and HMS 18046 (both belonging to the Haarlem family) share a mutation in the second base of codon 2 after the ribosomal slippery (overlapping region, Asp2Gly). HMS 18014 (T5_MAD2 family) has a mutation in the second base of codon 67 (*orfA*, Thr67Iso). As we have commented above, HCU 3717 (X family) has its two IS copies inserted in the DR region mutated (*orfB*, Gly215Ser). The rest of the strains studied have the wild-type IS.

Among all the copies studied, we found two different mutations for three strains in the IR of the IS*6110* (Figure 2): a SNP in base 6 of the IR flanking *orfA*, shared by HMS 1693 and HMS 14017 strains (L6) in the copy inserted in *lipX:mshB*; and a different SNP in base 9 of the IR flanking the *orfB*, in the copy inserted in *Rv0963c* of the HMS 18037 Haarlem strain.

### Study of IS*6110* Copies in *ppe* Genes

**Figure 3:**
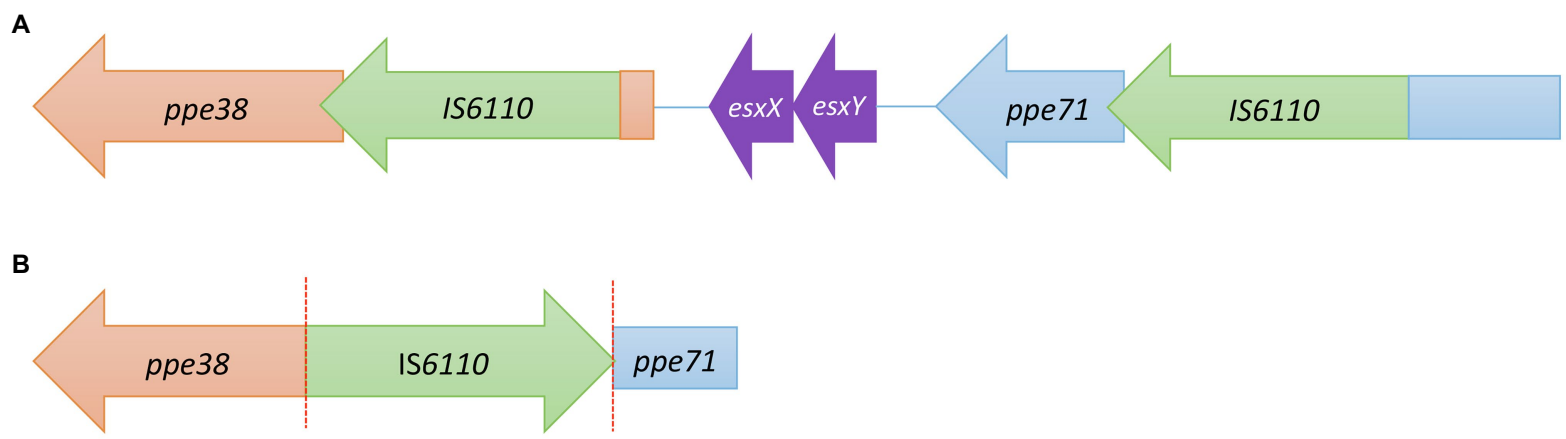
**(A)** Configuration of the *ppe38* locus in the MtZ strain. **(B)** Configuration of the *ppe38* locus in the Beijing GC1237 strain. The red lines indicate a truncation of the gene.

PPE regions are hot spots for the IS*6110* insertion (Sampson et al., 1999; Beggs et al., 2000), generating a high variability in the genome. In addition, these genes are difficult to amplify because the high number of repetitive regions and a high GC content. Nevertheless, we were successful in the amplification of two IS copies located in *ppe38* and *ppe71* for the MtZ strain (−) and one copy located between *ppe38/ppe71* (+) in Beijing GC1237 (Figure 3). This IS in the Beijing GC1237 strain produced a deletion of *esx* genes and the truncation of both *ppe* genes. We were also successful in the amplification of an IS*6110* copy located downstream of the *ppe46* gene, between the *pe27a* and *esxR* genes. In addition, we amplified another IS*6110* inserted within *ppe34*. We observed a different organisation of this gene compared with the H37Rv strain, with short repeated fragments of *ppe34* after the IS, a feature that may be typical of the Beijing family (H37Rv does not have these repetitions), as using the BLAST tool of the NCBI; Beijing genomes were the full matches. As we stated before, these copies do not have mutations.

## Discussion

This work deepens the knowledge of the IS*6110* sequence in the MTBC. IS*6110* has been used for a long time for diagnosis and genotyping, but very little is known about its genetic variability, mainly due to the difficulties in determining its complete sequence for each copy location in the genome. The limited data that have been published regarding this issue have shown dissimilar results (Dale et al., 1997; Thabet et al., 2015).

We have analysed the sequences of both *orfA* and *orfB* genes that constitute the transposase in several strains for which we knew its exact location in the genome using specific primers for the amplification of each copy. So far, the only data reported on the sequence of IS*6110* were obtained using internal primers that interchangeably amplify all the IS copies of the genome (Thabet et al., 2015), or the number of analysed copies was really low (Dale et al., 1997). In addition, as only one copy of each strain was analysed, the intra-strain variability was not studied. Therefore, the novelty of this work, as far as we know, is that for the first time the IS*6110* sequence corresponding to each location has been identified and sequenced, and likewise, the sequences of the different copies in the same strain or among families have been compared. Furthermore, IS*6110* cannot be studied directly using the most frequent whole genome sequencing (WGS) technologies, as is the case of Illumina, due to repetitive sequences are not correctly aligned; thus, this study complements this handicap.

We studied 158 IS*6110* sequences from 55 different strains belonging to L2 (Beijing), L3 (CAS_DELHI), L4 (Haarlem, LAM, T, S, and X), L6, and the animal branch, including strains with a high and a LCN of IS*6110*. There were variations of the wild-type sequence in 21 (13.3%) of the copies belonging to 16 strains (29%). We observed a higher percentage of mutated copies among the LCN strains (31.1 vs. 6.2% in high copy number), which could be related with a less transposition ability. To support this idea, a higher number of copies with a widely strain spectrum should be studied.

We found 10 different mutations (Figure 2). Five of them are located in the *orfB* of the transposase vs. two in the *orfA*, findings that seem to indicate that *orfB* is more susceptible to mutation. A possible explanation could be that the *orfB* is three times longer than *orfA*. One mutation is in the overlapping region affecting both ORFs. In addition, two other mutations are in the IR, which we do not expect to be involved in the transposase function as they are outside the coding region. Thabet et al. (2015) also found more polymorphic sites for *orfB* than for *orfA*. They suggested that non-synonymous mutations tend to be eliminated because the ancestral IS*6110* copy inherited by the MTBC would be functionally optimal. However, we found five out of eight mutations in the coding *orfA* and *orfB* that are non-synonymous. The fact that some of them are conserved in different strains supports the idea that those non-synonymous mutations are neutral in these cases, as Thabet et al. (2015) suggested. They described different haplotypes for *orfA* and *orfB*, reporting the wild type as the most frequent, similarly to our findings. On the other hand, our results differed from Thabet et al. (2015) in other aspects, including the description of some haplotypes with 1–3 SNPs. This caught our attention because all the variant copies we found have just one SNP. The high variability found by these authors could be partially caused by the different procedures applied to obtain the IS*6110* DNA sequence. We used specific primers for each location, what allowed us to sequence directly, for each copy, the DNA obtained from PCR. In contrast, other authors used IS internal primers to amplify, clone and sequence one of the copies of each strain without knowing what copy had been amplified or if the sequence obtained was the sum of several. One of the mutations they found (haplotype 20 of *orfB*) is Gly215Ser (*orfB*), the same we found in the X family. Since their collection did not include any X strains, we conclude that this mutation is not restricted to the X family. None of the other mutations obtained matched their results. Our results completely differed from Dale et al. (1997), who did not find any mutation when comparing five IS copies of different *M. tuberculosis* strains, so they affirmed that the IS has been totally conserved among the MTBC. Surely, the low number of copies they studied caused these differences.

As far as we know, this is the first time that all the IS copies of a strain have been studied. In some cases, we observed that it exists variability among the IS*6110* copies of the same strain, finding wild-type and mutated copies at the same time. In addition, we did not find copies with different mutations in the same strain.

Our expertise in genotyping *M. tuberculosis* by IS*6110*-RFLP supports the fact, previously described (Gonzalo-Asensio et al., 2018), that modern lineages (L2, L3, and L4) have accumulated more copies of IS*6110* in their genomes than ancient lineages (L1, L5, and L6). Modern lineages are better adapted to high-density populations, so it is possible that the number of ISs is related to this success (Gonzalo-Asensio et al., 2018). This eventuality agrees with the obtained results because the two most successful strains in our population, used in this work, are high copy number strains (MtZ and GC1237) and, curiously, all their copies (12 and 18, respectively) are wild type. Remarkably, all the strains of the X family (L4) studied, such as CDC1551; share a characteristic variant in at least one of their IS*6110* copies. According to these results, it is possible that this mutation could affect the transposition ability of IS*6110*, reducing its transposition frequency. Thereby, there would be a greater probability that the wild-type copies transpose, leading to a higher copy number of IS*6110* in the genome. This phenomenon could partially explain why the X family strains, belonging to L4, are LCN strains despite being a successful clinical family. Nevertheless, the mutation found in the X family strains did not prevent the transposition, as there are more copies with the same SNP in the same strain. Dale et al. (1997) suggested that the differences observed in the copy number are not related to structural changes in the transposase, but rather to the genomic region in which it is inserted. In view of our results, focusing especially on the X strains, we think that both facts are not mutually exclusive and more than one factor must be affecting its transposition.

It is notable that the Haarlem and X family strains studied have an IS*6110* inserted in *Rv0403c* at the same point and direction (483,296, −), in addition to an identical 3 bp created duplication. This finding suggests a common origin for both families, supporting all the phylogenetic trees in which the X and Haarlem families are closely related (Stucki et al., 2016). All the X strains studied in this work have a mutated copy located in *Rv0403c*, unlike the Haarlem strains, which lack a mutation. The evolutionary process that is inferred by these results is that the common ancestor of these two families had the IS*6110* copies located in the DR region and in the *Rv0403c* gene. Subsequently, the mutation in the copy located in *Rv0403c* of the X strains occurred after the phylogenetic separation between the Haarlem and X families, and this mutated copy was the one that transposed and led to the rest of the copies in this family, because all IS*6110* copies have the same mutation, except the one located in the DR region. The exception is HCU 3717, which has its two DR copies also mutated, suggesting a different evolutionary process.

Researchers demonstrated that the synthesis of the transposase is regulated post-transcriptionally by the ribosome (Gonzalo-Asensio et al., 2018). *orfA* finishes on Leu92 (UUA), but the ribosome can make a −1 frameshift and starts *orfB* with Lys1 (AAA). Considering this, we conclude that the mutation in the IS sequences of the X family strains has changed glycine 215 for serine (*orfB*). Glycine is the smallest amino acid and the only one that is not chiral. Furthermore, it could provide more flexibility to the protein region. On the contrary, serine is a polar amino acid, uncharged, and is bigger than glycine, a factor that diminishes the flexibility of the protein chain. We believe it is possible that this amino acid change may affect the protein structure but, according to our results, it does not prevent the transposition ability.

Regarding *M. africanum*, in both NCBI strains one of its IS*6110* copies is mutated. While the mutation found in the GM041182 strain is synonymous, strain 25 has a gap in the sequence, which completely alters the reading frame and probably disrupts the transposase. Among the LCN strains we sequenced, isolated in our region, *M. africanum* HMS 2382 has a non-synonymous mutation in one of its three IS*6110* copies. It changes aspartic acid for glycine, an important change because aspartic acid has negative charge and is larger than glycine. Although the Haarlem family is a completely different lineage (L4) than *M. africanum* (L6), we found that two Haarlem strains (HMS 18009 and HMS 18046) share the same mutation as HMS 2382, in the same nucleotide and in the same IS copy (DR region). In addition, IS*6110* has been inserted at exactly the same point in the three strains. As both L6 and L4 are separate phylogenetic branches, it is not likely that they acquired the mutation from a common ancestor; therefore, the mutation could be random. There is one mutation in the IR of the IS*6110* for HMS 1693 and HMS 14017 (L6). These two strains seem to be from the same cluster according to their RFLP patterns, a finding that would be supported by this shared mutation. As it is out of the coding region, we do not know if it affects the transposase somehow.

Among the findings in the other MTBC families studied is the mutation in the IS*6110* copy of the DR region in HMS 18014 (T family), which changes threonine to isoleucine. Both amino acids have a similar size, but they are in different classification groups. Threonine has a polar lateral chain without a charge, whereas isoleucine has a hydrophobic lateral chain, so the interactions will be different. Notably, the three Haarlem strains studies have a different mutation in the copy located in *Rv0963c*, one with a mutation in the IR of the IS (HMS 18037) and two with a synonymous mutation in the coding region of the transposase (HSJ 234 and HMS 18031). These mutations seem to be random as they are not the same or conserved among strains. The chance of three different mutations in the same location could be because a high number of these copies were sequenced. HMS 18025 (LAM) has a mutation in the copy inserted in *lpqQ:Rv0836c*, with a change from glycine to arginine. This is an important structural change because arginine is larger than glycine and has a positive charge. More studies are needed to determine how these mutations affect the transposition ability.

In total, we studied 38 IS copies located in the DR region. We successfully amplified this copy in 30 strains of our collection and examined eight more from the NCBI strains. Among them, we have found three different variants in five strains, described above. This is the copy in which we have found the most mutations, a phenomenon that can be explained because this is the region for which more IS copies have been studied, even though the percentage of mutation is constant with regard to the total mutations found. As almost all MTBC strains have an IS copy in this region, it would be reasonable to think that this copy could have been the first IS*6110* that started to transpose and thus has increased its number within the genome (McEvoy et al., 2007). If this is true, we have to assume that mutations in this copy occurred after the transposition, as the other studied copies do not have any mutations. Again, the exception is HCU 3717 (X family), in which all its IS*6110* copies, including the two of the DR region, seems to be mutated (in the absence of knowing the genotype of the copy located in *ppe46*).

Ates et al. (2018a) showed that modern Beijing families have different deletions involving the *ppe38* locus, suggesting that these changes may contribute to a higher growth rate *in vivo* and lung inflammation due to the block of PE_PGRS secretion, rendering some Beijing strains hypervirulent. The most virulent deletion described in their work was the one that disrupts the *ppe38* and *ppe71* genes with the loss of the *esx* genes between *ppe38* and *ppe71*. This is the deletion we found in the Beijing GC1237 strain (Figure 3). We found that this IS copy has no direct repeats, which suggests that there has been a recombination event between two I*S6110* copies with the removal of the genes between them (Brosch et al., 1999; Sampson et al., 2003). Regarding the MtZ strain, there is an IS*6110* within the *ppe38* gene and another one in the *ppe71* gene. We do not know how the insertion affects both genes. Ates et al. (2018b) demonstrated that a single copy of these *ppe* genes is enough to support PE_PGRS secretion; therefore, MtZ could have this secretion affected. Several experiments are ongoing to determine whether the secretion of PE_PGRS in the MtZ strain is altered. McEvoy et al. (2009) found IS*6110* insertion events in the *ppe38* locus in other MTBC families. As we also found this in the MtZ strain, we conclude that it is not something specific to the Beijing family. As we have stated previously, none of the IS*6110* copies amplified that are inserted in *ppe* genes have a mutation.

In summary, we have studied the genetic variability of IS*6110*. We have analysed the DNA sequence of the different IS*6110* copies in the same strain of *M. tuberculosis* as well as in different MTBC strains. We have observed mutation in 13.3% of the copies studied. Gly215Ser (*orfB*) mutation seems to be characteristic of the X family. In general, the high copy number strains analysed carry wild-type copies whereas several LCN strains, such as the X family and L6, have one or more of their copies mutated. Some strains share the location as well as mutations in these copies, allowing us in these cases to establish the transposition events that have occurred over time and indicating a close relationship in the evolution of these strains. The detailed study of each of the copies has also allowed us to provide information regarding the evolution of some MTBC families. Many publications have studied the variability among strains of the MTBC caused by changes in the number and location of IS*6110*. To these, we add the changes produced over time in the sequence itself as one more factor that will probably affect its evolution.

## Data Availability Statement

The datasets presented in this study can be found in online repositories. The names of the repository/repositories and accession number(s) can be found at: https://www.ncbi.nlm.nih.gov/; MZ574181–MZ574188.

## Author Contributions

SS and IO: conceptualised the work. JC: carried out the laboratory experiments and curated the data. JC, IO, and SS: wrote the manuscript. All authors contributed to the article and approved the submitted version.

## Funding

The author(s) declare financial support was received for the research, authorship, and/or publication of this article. This work was supported by the Carlos III Health Institute (ISCIII), AES2018 program, in the context of a Grant (FIS18/0336), co-financed by European Regional Development Funds of the European Commission: “A way of making Europe.” JC was awarded a scholarship by the Government of Aragon/European Social Fund, “Building Europe from Aragon.”

## Conflict of Interest

The authors declare that the research was conducted in the absence of any commercial or financial relationships that could be construed as a potential conflict of interest.

## Publisher’s Note

All claims expressed in this article are solely those of the authors and do not necessarily represent those of their affiliated organizations, or those of the publisher, the editors and the reviewers. Any product that may be evaluated in this article, or claim that may be made by its manufacturer, is not guaranteed or endorsed by the publisher.

## Footnotes

^1^ http://genolist.pasteur.fr/TubercuList/

^2^ http://genolist.pasteur.fr/BoviList/

^3^ https://www.ncbi.nlm.nih.gov/

## References

[ref1] Alonso H. Aguilo J. I. Samper S. Caminero J. A. Campos-Herrero M. I. Gicquel B.. (2011). Deciphering the role of IS6110 in a highly transmissible *Mycobacterium tuberculosis* Beijing strain, GC1237. Tuberculosis 91, 117–126. doi: 10.1016/j.tube.2010.12.007, PMID: 21256084

[ref2] Ates L. S. Dippenaar A. Ummels R. Piersma S. R. Van Der Woude A. D. Van Der Kuij K.. (2018a). Mutations in ppe38 block PE-PGRS secretion and increase virulence of *Mycobacterium tuberculosis*. Nat. Microbiol. 3, 181–188. doi: 10.1038/s41564-017-0090-6, PMID: 29335553

[ref3] Ates L. S. Sayes F. Frigui W. Ummels R. MPM D. Bottai D.. (2018b). RD5-mediated lack of PE_PGRS and PPE-MPTR export in BCG vaccine strains results in strong reduction of antigenic repertoire but little impact on protection. PLoS Pathog. 14:e1007139. doi: 10.1371/journal.ppat.1007139, PMID: 29912964 PMC6023246

[ref4] Beggs M. L. Eisenach K. D. Cave M. D. (2000). Mapping of IS6110 insertion sites in two epidemic strains of *Mycobacterium tuberculosis*. J. Clin. Microbiol. 38, 2923–2928. doi: 10.1128/JCM.38.8.2923-2928.2000, PMID: 10921952 PMC87149

[ref5] Brisson-Noel A. Aznar C. Chureau C. Nguyen S. Pierre C. Bartoli M.. (1991). Diagnosis of tuberculosis by DNA amplification in clinical practice evaluation. Lancet 338, 364–366. doi: 10.1016/0140-6736(91)90492-8, PMID: 1677709

[ref6] Brosch R. Philipp W. J. Stavropoulos E. Colston M. J. Cole S. T. Gordon S. V. (1999). Genomic analysis reveals variation between *Mycobacterium tuberculosis* H37Rv and the attenuated *M. tuberculosis* H37Ra strain. Infect. Immun. 67, 5768–5774. doi: 10.1128/IAI.67.11.5768-5774.1999, PMID: 10531227 PMC96953

[ref7] Comín J. Cebollada A. Ibarz D. Viñuelas J. Vitoria M. A. Iglesias M. J.. (2021). A whole-genome sequencing study of an X-family tuberculosis outbreak focus on transmission chain along 25 years. Tuberculosis 126:102022. doi: 10.1016/j.tube.2020.102022, PMID: 33341027

[ref8] Comin J. Chaure A. Cebollada A. Ibarz D. Viñuelas J. Vitoria M. A.. (2020). Investigation of a rapidly spreading tuberculosis outbreak using whole-genome sequencing. Infect. Genet. Evol. 81:104184. doi: 10.1016/j.meegid.2020.104184, PMID: 31931260

[ref9] Comín J. Monforte M. L. Samper S. Otal I. (2021). Analysis of *Mycobacterium africanum* in the last 17 years in Aragon identifies a specific location of IS6110 in lineage 6. Sci. Rep. 11:10359. doi: 10.1038/s41598-021-89511-x, PMID: 33990628 PMC8121931

[ref10] Dale J. W. (1995). Mobile genetic elements in mycobacteria. Eur. Respir. J. Suppl. 20, 633s–648s. PMID: 8590564

[ref11] Dale J. W. Tang T. H. Wall S. Zainuddin Z. F. Plikaytis B. (1997). Conservation of IS6110 sequence in strains of *Mycobacterium tuberculosis* with single and multiple copies. Tuber. Lung Dis. 78, 225–227. doi: 10.1016/S0962-8479(97)90002-2, PMID: 10209676

[ref12] Fang Z. Doig C. Morrison N. Watt B. Forbes K. J. (1999). Characterization of IS1547, a new member of the IS900 family in the *Mycobacterium tuberculosis* complex, and its association with IS6110. J. Bacteriol. 181, 1021–1024. doi: 10.1128/JB.181.3.1021-1024.1999, PMID: 9922269 PMC93472

[ref13] Fang Z. Forbes K. J. (1997). A *Mycobacterium tuberculosis* IS6110 preferential locus (ipl) for insertion into the genome. J. Clin. Microbiol. 35, 479–481. doi: 10.1128/jcm.35.2.479-481.1997, PMID: 9003621 PMC229605

[ref14] Gonzalo-Asensio J. Pérez I. Aguiló N. Uranga S. Picó A. Lampreave C.. (2018). New insights into the transposition mechanisms of IS6110 and its dynamic distribution between *Mycobacterium tuberculosis* complex lineages. PLoS Genet. 14:e1007282. doi: 10.1371/journal.pgen.1007282, PMID: 29649213 PMC5896891

[ref15] Hermans P. W. M. Van Soolingen D. Bik E. M. De Haas P. E. W. Dale J. W. Van Embden J. D. A. (1991). Insertion element IS987 from Mycobacterium bovis BCG IS located in a hot-spot integration region for insertion elements in *Mycobacterium tuberculosis* complex strains. Infect. Immun. 59, 2695–2705. doi: 10.1128/iai.59.8.2695-2705.1991, PMID: 1649798 PMC258075

[ref16] Isabel Millan-Lou M. Isabel López-Calleja A. Colmenarejo C. Antonia Lezcano M. Asunción Vitoria M. Del Portillo P.. (2013). Global study of is6110 in a successful *Mycobacterium tuberculosis* strain: clues for deciphering its behavior and for its rapid detection. J. Clin. Microbiol. 51, 3631–3637. doi: 10.1128/JCM.00970-13, PMID: 23985924 PMC3889744

[ref17] McEvoy C. R. E. Falmer A. A. van Pittius N. C. G. Victor T. C. van Helden P. D. Warren R. M. (2007). The role of IS6110 in the evolution of *Mycobacterium tuberculosis*. Tuberculosis 87, 393–404. doi: 10.1016/j.tube.2007.05.010, PMID: 17627889

[ref18] McEvoy C. R. Van Helden P. D. Warren R. M. Van Pittius N. C. G. (2009). Evidence for a rapid rate of molecular evolution at the hypervariable and immunogenic *Mycobacterium tuberculosis* PPE38 gene region. BMC Evol. Biol. 9, 237–221. doi: 10.1186/1471-2148-9-237, PMID: 19769792 PMC2758852

[ref19] Mendiola M. V. Martin C. Otal I. Gicquel B. (1992). Analysis of the regions responsible for IS6110 RFLP in a single *Mycobacterium tuberculosis* strain. Res. Microbiol. 143, 767–772. doi: 10.1016/0923-2508(92)90104-V, PMID: 1363676

[ref20] Otal I. Martin C. Vincent-Levy-Frebault V. Thierry D. Gicquel B. (1991). Restriction fragment length polymorphism analysis using IS6110 as an epidemiological marker in tuberculosis. J. Clin. Microbiol. 29, 1252–1254. doi: 10.1128/jcm.29.6.1252-1254.1991, PMID: 1677943 PMC269979

[ref21] Reyes A. Sandoval A. Cubillos-Ruiz A. Varley K. E. Hernández-Neuta I. Samper S.. (2012). IS-seq: a novel high throughput survey of in vivo IS6110 transposition in multiple *Mycobacterium tuberculosis* genomes. BMC Genomics 13:249. doi: 10.1186/1471-2164-13-249, PMID: 22703188 PMC3443423

[ref22] Robinson J. T. Thorvaldsdóttir H. Winckler W. Guttman M. Lander E. S. Getz G.. (2011). Integrative genome viewer. Nat. Biotechnol. 29, 24–26. doi: 10.1038/nbt.1754, PMID: 21221095 PMC3346182

[ref23] Roychowdhury T. Mandal S. Bhattacharya A. (2015). Analysis of IS6110 insertion sites provide a glimpse into genome evolution of *Mycobacterium tuberculosis*. Sci. Rep. 5, 1–10. doi: 10.1038/srep12567, PMID: 26215170 PMC4517164

[ref24] Safi H. Barnes P. F. Lakey D. L. Shams H. Samten B. Vankayalapati R.. (2004). IS6110 functions as a mobile, monocyte-activated promoter in *Mycobacterium tuberculosis*. Mol. Microbiol. 52, 999–1012. doi: 10.1111/j.1365-2958.2004.04037.x, PMID: 15130120

[ref25] Sagasti S. Millán-Lou M. I. Soledad Jiménez M. Martín C. Samper S. (2016). In-depth analysis of the genome sequence of a clinical, extensively drug-resistant *Mycobacterium bovis* strain. Tuberculosis 100, 46–52. doi: 10.1016/j.tube.2016.06.005, PMID: 27553409

[ref26] Sampson S. L. Warren R. M. Richardson M. van der Spuy G. D. van Helden P. D. (1999). Disruption of coding regions by IS6110 insertion in *Mycobacterium tuberculosis*. Tuber. Lung Dis. 79, 349–359. doi: 10.1054/tuld.1999.0218, PMID: 10694979

[ref27] Sampson S. L. Warren R. M. Richardson M. Victor T. C. Jordaan A. M. Van der Spuy G. D.. (2003). IS6110-mediated deletion polymorphism in the direct repeat region of clinical isolates of *Mycobacterium tuberculosis*. J. Bacteriol. 185, 2856–2866. doi: 10.1128/JB.185.9.2856-2866.2003, PMID: 12700265 PMC154393

[ref28] Sekine Y. Izumi K. I. Mizuno T. Ohtsubo E. Ishihama A. (1997). Inhibition of transpositional recombination by OrfA and OrfB proteins encoded by insertion sequence IS3. Genes Cells 2, 547–557. doi: 10.1046/j.1365-2443.1997.1440342.x, PMID: 9413996

[ref29] Soto C. Y. Menéndez M. C. Pérez E. Samper S. Gómez A. B. García M. J.. (2004). IS6110 mediates increased transcription of the phoP virulence gene in a multidrug-resistant clinical isolate responsible for tuberculosis outbreaks. J. Clin. Microbiol. 42, 212–219. doi: 10.1128/JCM.42.1.212-219.2004, PMID: 14715755 PMC321672

[ref30] Stucki D. Brites D. Jeljeli L. Coscolla M. Liu Q. Trauner A.. (2016). *Mycobacterium tuberculosis* lineage 4 comprises globally distributed and geographically restricted sublineages. Nat. Genet. 48, 1535–1543. doi: 10.1038/ng.3704, PMID: 27798628 PMC5238942

[ref31] Supply P. Allix C. Lesjean S. Cardoso-Oelemann M. Rüsch-Gerdes S. Willery E.. (2006). Proposal for standardization of optimized mycobacterial interspersed repetitive unit-variable-number tandem repeat typing of *Mycobacterium tuberculosis*. J. Clin. Microbiol. 44, 4498–4510. doi: 10.1128/JCM.01392-06, PMID: 17005759 PMC1698431

[ref32] Tanaka M. M. (2004). Evidence for positive selection on *Mycobacterium tuberculosis* within patients. BMC Evol. Biol. 4, 31–38. doi: 10.1186/1471-2148-4-31, PMID: 15355550 PMC518962

[ref33] Thabet S. Namouchi A. Mardassi H. (2015). Evolutionary trends of the transposase-encoding open reading frames A and B (orfA and orfB) of the mycobacterial IS6110 insertion sequence. PLoS One 10:e0130161. doi: 10.1371/journal.pone.0130161, PMID: 26087177 PMC4473070

[ref34] Thierry D. Brisson-Noel A. Vincent-Levy-Frebault V. Nguyen S. Guesdon J. L. Gicquel B. (1990). Characterization of a *Mycobacterium tuberculosis* insertion sequence, IS6110, and its application in diagnosis. J. Clin. Microbiol. 28, 2668–2673. doi: 10.1128/jcm.28.12.2668-2673.1990, PMID: 2177747 PMC268253

[ref35] Vera-Cabrera L. Hernández-Vera M. A. Welsh O. Johnson W. M. Castro-Garza J. (2001). Phospholipase region of *Mycobacterium tuberculosis* is a preferential locus for IS6110 transposition. J. Clin. Microbiol. 39, 3499–3504. doi: 10.1128/JCM.39.10.3499-3504.2001, PMID: 11574563 PMC88379

[ref36] Wall S. Ghanekar K. McFadden J. Dale J. W. (1999). Context-sensitive transposition of IS6110 in mycobacteria. Microbiology 145, 3169–3176. doi: 10.1099/00221287-145-11-3169, PMID: 10589725

[ref37] Warren R. M. Sampson S. L. Richardson M. Van Der Spuy G. D. Lombard C. J. Victor T. C.. (2000). Mapping of IS6110 flanking regions in clinical isolates of *Mycobacterium tuberculosis* demonstrates genome plasticity. Mol. Microbiol. 37, 1405–1416. doi: 10.1046/j.1365-2958.2000.02090.x, PMID: 10998172

[ref38] Yesilkaya H. Dale J. W. Strachan N. J. C. Forbes K. J. (2005). Natural transposon mutagenesis of clinical isolates of *Mycobacterium tuberculosis*: how many genes does a pathogen need? J. Bacteriol. 187, 6726–6732. doi: 10.1128/JB.187.19.6726-6732.2005, PMID: 16166535 PMC1251597

