# Corrigendum: In-depth analysis of IS*6110* genomic variability in the *Mycobacterium tuberculosis* complex

**DOI:** 10.3389/fmicb.2025.1544186

**Published:** 2025-01-31

**Authors:** Jessica Comín, Isabel Otal, Sofía Samper

**Affiliations:** ^1^Unidad de Investigación Traslacional, Hospital Universitario Miguel Servet, Instituto Aragonés de Ciencias de la Salud, Zaragoza, Spain; ^2^Fundación IIS Aragón, Zaragoza, Spain; ^3^Facultad de Medicina, Universidad de Zaragoza, Zaragoza, Spain; ^4^CIBER de Enfermedades Respiratorias, Madrid, Spain

**Keywords:** tuberculosis, IS*6110*, *Mycobacterium tuberculosis* complex, IS*6110* genomic variability, tuberculosis evolution

In the published article, there was an error in the **Funding statement**. The article should contain the name of FEDER, the European Regional Development Funds of the European Commission, and the sentence: “A way of making Europe”.

Original Text: This work was supported by the Carlos III Health Institute in the context of a Grant (FIS18/0336), and JC was awarded a scholarship by the Government of Aragon/European Social Fund, “Building Europe from Aragon.” The correct Funding statement appears below.

